# Scoliosis and Gastroesophageal Reflux Disease in Adults

**DOI:** 10.7759/cureus.15359

**Published:** 2021-05-31

**Authors:** Fahri Eryilmaz, Faheem Ahmed, Asim K Rehmani, Sundas Karimi, Aamna Qazi, Sufyan Mustafa, Arif Zulfiqar, Zubia Nadeem, Ayyaz A Sultan, Umar Farooque

**Affiliations:** 1 Neurological Surgery, Hitit University Corum Erol Olcok Training and Research Hospital, Corum, TUR; 2 Orthopedic Surgery, Trauma Centre, Civil Hospital, Karachi, PAK; 3 Neurological Surgery, National Medical Center, Karachi, PAK; 4 Orthopedic Surgery, Dow University Hospital, Karachi, PAK; 5 Medicine, Dow University of Health Sciences, Karachi, PAK; 6 Medicine, Dow Medical College, Civil Hospital, Karachi, PAK; 7 Medicine and Surgery, Dow Medical College, Karachi, PAK; 8 Hematology/Oncology, California Cancer Associates for Research and Excellence, Fresno, USA; 9 Neurology, Dow University of Health Sciences, Karachi, PAK

**Keywords:** gastroesophageal reflux disease, scoliosis, quest score, cobb angle, sagittal plane

## Abstract

Introduction

Degenerative scoliosis most commonly presents with lower back pain. Literature suggests that adults who have degenerative scoliosis are at greater risk of both hiatal hernia and gastroesophageal reflux disease (GERD). The objective of this study was to evaluate scoliosis as being the risk factor of GERD in adults.

Materials and methods

This prospective study was conducted at Dow University of Health Sciences over a period of two years (May 2018 to April 2020). The investigation included 210 participants with spinal disorders. The mean age was 71.6±9.6 years. The X-rays of the participants’ whole spine were taken in a standing position, in the sagittal and coronal planes. Symptoms of GERD were measured through the quality of life and utility evaluation survey technology (QUEST) score, taking six points as cutoff values. The evaluation was done using radiographs to determine any relationship between spinal disorders and GERD. Negative values were analyzed in a right-sided convex curve while positive values in the left-sided convex curve were viewed in the coronal plane. Degenerative scoliosis was explained as a lumbar/thoracolumbar Cobb angle of more than 10 degrees. Univariate and multivariate logistic regression analyses were done to assess the risk factors related to GERD.

Results

Out of 210 patients, 146 were found to have degenerative scoliosis at the level of the lumbar and thoracolumbar spine. Fifty-two patients had a right convex curve, and 94 had a left convex curve. Sixty-nine patients had GERD. According to the analysis of the multivariate logistic regression, the Cobb angle was highly related to GERD (p-value <0.05 and odds ratio of 1.031). The participants were grouped according to the Cobb angle of curve at the lumbar spine (less than 30 degrees with a large right-sided convex curve, 30 and more with a small curve, and more than 30+ degrees with a large left-sided convex curve). The study revealed that a large left-sided convex curve was highly related to GERD, with a p-value <0.05 and odds ratio of 10.935.

Conclusions

The left-sided large convex curve at the thoracolumbar or lumbar spine, especially when the Cobb angle was more than 30 degrees, was highly associated with GERD. Therefore, the symptoms of GERD should be monitored in the elderly population with degenerative scoliosis.

## Introduction

Lower back pain is a common presentation in patients with degenerative scoliosis. Other symptoms such as leg pain, improper balance in the sagittal and coronal planes, and neurological deficits may appear as well [1,2]. It can be elucidated as a Cobb angle of more than 10 degrees in the coronal plane in patients with a mature skeleton [3]. According to a study in the United States, approximately 8.9% of individuals aged 40 years were found to have lumbar scoliosis, which showed a close relationship between scoliosis and age. The risk of having scoliosis increases from the fifth to the sixth decade of life, with a high prevalence in individuals more than 90 years of age [4,5]. Therefore, spinal deformity can be expected in elderly populations. The spinal deformity can cause both musculoskeletal (MSK) and viscera-related disorders, especially in organs near the spine [6,7]. The patients with severe scoliosis at the thoracic region are more prone to problems associated with the pulmonary system, such as reduced total lung capacity (TLC) and decreased forced vital capacity (FVC), which are highly associated with idiopathic scoliosis >40 degrees and kyphosis >50 degrees [8].

The adults who have a degenerative spine with sagittal deformities such as lumbar kyphosis or lumbar and thoracic vertebral fractures were found to be at greater risk of hiatal hernia and gastroesophageal reflux disease (GERD) [9]. Defined as the backflow of gastric and duodenal contents into the esophagus, GERD causes acid regurgitation and heartburn. According to Bari et al., the symptoms of GERD are highly affected by the muscular strength of the back, the lordosis angle of the lumbar, and sagittal balance [10]. The pathophysiology behind GERD determines the impaired functions related to the mechanisms of anti-reflux such as esophageal peristalsis, the lower esophageal sphincter, the pressure gradient of the thoracoabdominal region, and diaphragmatic impairment [10]. Because of the weakened lower esophageal sphincter that allows the retrograde flow of contents into the esophagus, hiatal hernias also cause GERD. The reflux symptoms are prevalent in approximately 10-20% of the European and North American population and 6.5-9.5% of the population in Japan. In addition, the risk of having GERD increases with aging [9,11]. It is prevalent in approximately 37.6% of the population of Japan, according to the questionnaire frequency scale for the symptoms of gastroesophageal reflux disease (FSSG) and the quality of life and utility evaluation survey technology (QUEST) [9]. However, the relation between GERD and scoliosis was not analyzed in adults. Therefore, the goal of our study was to assess the effects of truncal deformity on GERD in adults.

## Materials and methods

This prospective study was conducted at Dow University of Health Sciences for a period of two years, from May 2018 to April 2020. The participants of age 40 years or more and both with and without spinal deformity took part in the study. The patients with spinal deformity underwent a radiographic investigation of the whole spine, in the standing position in both the coronal and the sagittal planes. The data were collected using the QUEST questionnaire. About 246 patients underwent a spinal X-ray and gave QUEST results. Thirty-six participants were not included in the study; 29 were excluded because they gave incomplete data in QUEST, six were excluded due to their history of spinal surgery, and one was excluded because of taking oral steroids. None of the patients revealed any history of previous surgery of esophageal, duodenal, or gastric diseases. Almost 210 participants with a mean age of about 71.6±9.6 years and a range of about 45 to 85 years old were included; 61 were male and 149 were female. The diagnoses revealed degenerative lumbar spondylosis, kyphoscoliosis, lumbar stenosis, cervical myopathy, cervical degeneration, and spondylosis. The data regarding height, weight, and drug-related history were evaluated.

QUEST contains seven items to analyze the sensation felt by patients and as an association between the symptoms and exacerbation or relieving factors. The items were allocated according to negative, positive, or neutral scores, which were then added to get a range from seven to +18. The Japanese QUEST has specificity and sensitivity of 74% and 65%, respectively, with a 73% rate of accuracy when six points are set as cut-off values.

According to this study, the six-point QUEST score was considered a positive result of GERD. The radiographic evaluation was done in the lateral view, showing the thoracic vertebra fifth to 12th (T5-T12), the thoracolumbar region twelfth thoracic vertebra to the second lumbar spinal vertebra (T12-L2), and the lumbar lordosis angles at the level of the twelfth thoracic vertebra to sacral spinal nerve one (T12-S1). The pelvic tilt and incidence and the sacral slope and sagittal balance were noted. The lordosis was taken as a negative value and the kyphosis was taken as a positive value. The coronal balance was defined as the alignment of the spine between the C7 plumb line, and the central vertical line of the sacrum was noted in the frontal view. If the C7 plumb line was on the right side, the value was taken as positive; if it was on the left side, the value was taken as negative. The Cobb angle of the thoracolumbar and lumbar regions was noted. To differentiate the convex curves, the right-sided was taken as negative and the left-sided was taken as positive. Patients with a Cobb angle of more than 10 degrees were analyzed for degenerative scoliosis. The standard techniques were used to evaluate the radiographic results.

The statistical analysis was done using the Statistical Package for the Social Sciences (SPSS) version 21 (Armonk, NY: IBM Corp.), and the mean ± standard deviation (SD) of the data was evaluated. The Pearson or Spearman correlation coefficient analysis was carried out to determine the relationship between the QUEST and the radiographic analysis, which included the Cobb angle, trunk balance, and sagittal alignment. Using multivariate and univariate logistic regression analyses, the odds ratio was evaluated with a confidence interval of approximately 95% to check for the risk factors related to GERD. The p-value of ≤0.05 was taken, which was quite significant.

## Results

The mean values and the characteristics of the patients are given in Table 1. The mean Cobb angle value of 15.4±16.4 degrees was observed in all patients in the thoracic region and 24.6±21.6 in the lumbar region. Out of 136 patients who had lumbar degenerative scoliosis (curve >10 degrees), 52 had a right-sided convex curve with a Cobb angle <10 degrees, and 94 had a left-sided convex curve with a Cobb angle >10 degrees. The results revealed no such magnitude difference of the lumbar curve in both groups with curves in a different direction. About 64 participants had a Cobb angle <10 degrees, which was absolutely associated with a straight spine. The mean QUEST score in all patients was 4.7±5.3. GERD symptoms were found to be in 69 patients (32.8%), with the cut-off value set at six points.

**Table 1:** Demographic features of patients T5-T12: thoracic vertebra fifth to 12th; T12-L2: 12th thoracic vertebra to second lumbar spinal vertebra; T12-S1: 12th thoracic vertebra to sacral spinal nerve one; QUEST: quality of life and utility evaluation survey technology; GERD: gastroesophageal reflux disease

Variables	Values
Male/female	61/149
Age (years)	71.6±9.6
Thoracic Cobb angle (absolute value) (deg.)	15.4±16.4 (0-91.0)
Lumbar Cobb angle (absolute value) (deg.)	24.6±21.6 (0-100)
The mean of the right lumbar convex curve	52 cases
	(35.1±18.2 degrees)
The mean of the left lumbar convex curve	94 patients
	(+34.6±19.5 degrees)
Coronal balance (cm)	0.8±3.1
Thoracic kyphosis angle (T5-T12) (deg.)	24.6±16.6
Thoracolumbar kyphosis angle (T10-L2) (deg.)	17.2±17.4
Lumbar lordosis angle (T12-S1) (deg.)	27.2±21.1
Pelvic incidence (deg.)	52.3±11.3
Pelvic tilt (deg.)	25.4±12.2
Sacral slope (deg.)	27.9±10.4
Sagittal balance (cm)	6.7±6.3
QUEST score	4.7±5.3 (1-15)
GERD (+) (QUEST score Z6)	69 patients

The tested variables along with the QUEST scores are shown in Table 2. The analysis was done using the Pearson correlation coefficient. The QUEST scores were found to be related positively with the Cobb angle of the lumbar region with the correlation coefficient =0.270 and the Cobb angle of the thoracic region negatively correlated with the correlation coefficient =0.158. The p-value was taken as <0.05. The other variables or sagittal plane parameters such as lumbar lordosis, thoracolumbar kyphosis, and sagittal balance were associated with the QUEST score. The Spearman correlation analysis revealed correlations between the variables. It evaluated a strong relation between the Cobb angle of the thoracic region and the lumbar region with a correlation coefficient =0.858 and the relation between the sacral slope (SS) and lordosis of the lumbar spine with a correlation coefficient =0.762.

**Table 2:** Correlations between the QUEST score and variables (Pearson correlation analysis) QUEST: quality of life and utility evaluation survey technology

Variables	Correlation coefficient	p-Value
Sex	0.023	0.995
Age	0.115	0.156
Lumbar Cobb angle	0.270	<0.06
Thoracic Cobb angle	0.158	<0.06
Thoracic kyphosis angle	0.096	0.243
Coronal balance	0.061	0.493
Lumbar lordosis angle	0.059	0.507
Thoracolumbar kyphosis angle	0.033	0.762
Pelvic tilt	0.013	0.977
Pelvic incidence	0.029	0.9
Sagittal balance	0.06	0.59
Sacral slope	0.024	0.858

The univariate logistic regression analysis was carried out to evaluate the odds ratios of the risk factors associated with GERD (Table 3). The presence of the symptoms of GERD in the participants was considered as a dependent variable. The odds ratio of the coronal lumbar curve was found to be 1.031. It was 0.993 for the thoracic curve. The p-value was taken as <0.05.

**Table 3:** Univariate logistic regression analysis *Significant p-value. OR: odds ratio; CI: confidence interval; T5-T12: thoracic vertebra fifth to 12th; T12-S1: 12th thoracic vertebra to sacral spinal nerve one; T10-L2: 10th thoracic vertebra to second lumbar spinal vertebra

Variables	OR	95% CI	p-Value
Sex	1.106	0.556-2.211	0.806
Age	1.001	0.973-1.030	0.531
Lumbar Cobb angle	1.031*	1.020-1.042	<0.05
Thoracic Cobb angle	0.993*	0.978-1.008	<0.05
Thoracic kyphosis (T5-12)	1.001	0.981-1.021	0.376
Coronal balance	1.042	0.937-1.159	0.578
Lumbar lordosis (T12-S1)	1.019	1.003-1.034	0.281
Thoracolumbar kyphosis (T10-L2)	1.018	1.000-1.037	0.403
Pelvic tilt	1	0.983-1.038	0.938
Pelvic incidence	1.012	0.983-1.043	0.899
Sagittal balance	1.039	0.982-1.098	0.338
Sacral slope	1.011	0.979-1.045	0.949

Table 4 shows the multivariate logistic regression analysis. This analysis was done by excluding the thoracic Cobb angle and the SS, as they possess high rates of correlation coefficient with the Cobb angle and lordosis of the lumbar region.

**Table 4:** Multivariate logistic regression analysis *Significant p-value. OR: odds ratio; CI: confidence interval; T5-T12: thoracic vertebra fifth to 12th; T12-S1: 12th thoracic vertebra to sacral spinal nerve one; T10-L2: 10th thoracic vertebra to second lumbar spinal vertebra

Variables	OR	95% CI	p-Value
Lumbar Cobb angle	1.031*	1.020-1.043	<0.05
Sex	1.029	0.470-2.268	0.973
Age	1.001	0.969-1.034	0.561
Thoracic kyphosis (T5-12)	1.007	0.980-1.035	0.846
Coronal balance	0.989	0.882-1.109	0.727
Lumbar lordosis (T12-S1)	1.018	1.000-1.036	0.395
Thoracolumbar kyphosis (T10-L2)	1.018	0.996-1.040	0.492
Pelvic tilt	0.996	0.966-1.026	0.366
Sagittal balance	1.035	0.957-1.119	0.551
Pelvic incidence	1.036	0.985-1.089	0.342

The other variables do not show any correlations as shown in Table 5. The patients were categorized in accordance with the Cobb angle of the lumbar curve.

**Table 5:** Multivariate logistic regression analysis for GERD *Significant p-value. OR: odds ratio; CI: confidence interval; T5-T12: thoracic vertebra fifth to 12th; T10-L2: 10th thoracic vertebra to second lumbar spinal vertebra; T12-S1: 12th thoracic vertebra to sacral spinal nerve one; GERD: gastroesophageal reflux disease

Variables	OR	95% CI	p-Value
Lumbar Cobb angle (deg.) < 30	1		
Lumbar Cobb angle (deg.) ≥30	10.935*	3.261-53.799	<0.05
Age	1.002	1.959-2.026	0.647
Sex	1.06	1.426-3.160	0.931
Coronal balance	0.988	1.871-2.099	0.722
Thoracic kyphosis (T5-12)	1.004	1.967-2.022	0.691
Thoracolumbar kyphosis (T10-L2)	1.019	1.988-2.030	0.427
Lumbar lordosis (T12-S1)	1.019	1.991-2.027	0.333
Pelvic incidence	1.036	1.974-2.080	0.343
Pelvic tilt	0.998	1.957-2.019	0.442
Sagittal balance	1.031	1.946-2.103	0.601

## Discussion

The risk factors in causing GERD included smoking, hiatal hernia, overweight, alcohol consumption, negative Helicobacter pylori, and aging. Vertebral fractures or malalignments of the spine in the sagittal plane cause kyphosis, which is also associated with hiatal hernia sizes and GERD. The lumbar kyphosis and the vertebral fractures of the lumbar regions are highly related to GERD [12,13]. Although the lordosis of the lumbar spine, the muscular strength of the back, and sagittal balance are associated with GERD symptoms, the studies did not reveal the relation between deformities in the coronal plane and GERD [12].

Hiatal hernias can be associated with scoliosis in both the adolescent and the adult population. According to a study, approximately 6.2% of adolescents were observed as having hiatal hernia before surgery for idiopathic scoliosis, which was reduced to 2.1% after the surgery [14]. The analysis of another study indicated that coronal deformity might be associated with GERD because kyphosis is not common among patients with adolescent idiopathic scoliosis [15].

Even though all of the reports revealed that scoliosis might be associated with GERD, the skeletal evaluations noted from the spinal X-rays in standing were incomplete. Moreover, no study showed any significant relationship between the curve of scoliosis and the symptoms of GERD [16]. This study analyzed the data obtained by X-rays of the whole spine of patients in the frontal as well as the sagittal view in standing, which further assisted in the assessment of spinal deformities with accuracy, as compared with the spinal mouse or chest X-rays of the partial spinal view [17].

Although the sagittal plane parameters revealed positive and negative values in kyphosis and lordosis, respectively, the direction of the curve was not taken into consideration while evaluating the magnitude of the curve in scoliosis. Nonetheless, because of the left-sided location of the gastroesophageal junction, the difference between the right and the left-sided curves can be important in the evaluation of the relation between the symptoms of GERD and scoliosis [18]. Therefore, to analyze the direction of the curve when evaluating the risk factors of GERD, we explained the left-sided convex curve of the thoracolumbar or lumbar region as positive and the right-sided as negative.

This study successfully demonstrated that scoliosis of the thoracolumbar or lumbar region is associated with GERD. The odds ratio was 1.031 and the 95% confidence interval was 1.010-1.033, showing that the convex curve on the left side (positive value) is a significant risk factor of GERD. The Cobb angle categorized the curve with the left-sided curve more strongly associated with GERD (odds ratio {OR} = 10.935). The anatomical reasons revealed diaphragmatic changes that induce GERD due to the left-sided curve [19]. Because the curve apex may induce distortion of the esophageal hiatus, the attachment of the diaphragm on the spine at the thoracolumbar region may lead to hiatal hernia, which further leads to GERD [19].

While evaluating the spinal deformities in patients having hiatal hernia, in their study, Ushirozako et al. observed that 24% of the patients had scoliosis, and 12 out of 14 presented with the curve apex at the diaphragm. The left convex curve of the thoracolumbar or lumbar region may increase the pressure of the abdomen by abdominal spaces narrowing and pushing organs such as the stomach and the gastroesophageal junction, further causing acid regurgitation or hiatal hernia [20].

The Ishihara et al. study revealed the mechanism in which the reported case with left convex thoracolumbar neuromuscular kyphoscoliosis was analyzed by CT scan and the results showed the deviation of the axis to the left side between the esophagus and the stomach. The study further elaborated on the improvements in the symptoms of GERD after surgical management of scoliosis in that patient [21].

## Conclusions

The left-sided large convex curve at the thoracolumbar or lumbar was highly associated with GERD and the risk of having the disease increased when the Cobb angle was more than 30 degrees. Therefore, it was concluded that symptoms of GERD should be monitored in elderly patients who have degenerative scoliosis.

## References

[1] Ohba T, Ebata S, Oba H, Oda K, Tanaka N, Koyama K, Haro H (2020). Key radiographic parameters that influence the improvement of postoperative gastroesophageal reflux disease in patients treated surgically for adult spinal deformity with a minimum 2-year follow-up. Spine (Phila Pa 1976).

[2] Hasegawa T, Ushirozako H, Yamato Y (2020). Impact of adult spinal deformity corrective surgery in patients with the symptoms of gastroesophageal reflux disease: a 5-year follow-up report. Eur Spine J.

[3] Hori Y, Matsumura A, Namikawa T, Kato M, Iwamae M, Nakamura H (2021). Comparative study of the spinopelvic alignment in patients with idiopathic lumbar scoliosis between adulthood and adolescence. World Neurosurg.

[4] Miller F (2020). Cerebral palsy spinal deformity: etiology, natural history, and nonoperative management. Cerebral Palsy.

[5] Arima H, Hasegawa T, Yamato Y (2021). Factors associated with improved quality of life outcomes in patients undergoing surgery for adult spinal deformity. Spine (Phila Pa 1976).

[6] Imagama S, Ando K, Kobayashi K (2019). Increase in lumbar kyphosis and spinal inclination, declining back muscle strength, and sarcopenia are risk factors for onset of GERD: a 5-year prospective longitudinal cohort study. Eur Spine J.

[7] Ohba T, Ebata S, Oba H, Koyama K, Yokomichi H, Haro H (2019). Predictors of poor global alignment and proportion score after surgery for adult spinal deformity. Spine (Phila Pa 1976).

[8] Tanaka N, Ebata S, Oda K, Oba H, Haro H, Ohba T (2020). Predictors and clinical importance of postoperative coronal malalignment after surgery to correct adult spinal deformity. Clin Spine Surg.

[9] Ohba T, Oba H, Koyama K, Oda K, Tanaka N, Fujita K, Haro H (2020). Locomotive syndrome: prevalence, surgical outcomes, and physical performance of patients treated to correct adult spinal deformity. [In Press]. J Orthop Sci.

[10] Bari TJ, Hallager DW, Hansen LV, Dahl B, Gehrchen M (2021). Mechanical revision following pedicle subtraction osteotomy: a competing risk survival analysis in 171 consecutive adult spinal deformity patients. Spine Deform.

[11] Lee NJ, Lenke LG, Cerpa M (2020). The 90-day reoperations and readmissions in complex adult spinal deformity surgery. Global Spine J.

[12] Bari TJ, Hallager DW, Hansen LV, Dahl B, Gehrchen M (2021). Reducing revision rates following pedicle subtraction osteotomy surgery: a single-center experience of trends over 7 years in patients with adult spinal deformity. Spine Deform.

[13] Weigl DM (2019). Scoliosis in non-ambulatory cerebral palsy: challenges and management. Isr Med Assoc J.

[14] Tandon MS, Dhingra A, Varma V (2020). Management of patient with scoliosis. Problem Based Learning Discussions in Neuroanesthesia and Neurocritical Care.

[15] Nakashima H, Kanemura T, Satake K (2020). Lateral approach corpectomy and reconstruction after anterior longitudinal ligament release in cases with fixed kyphosis: a technical note and a preliminary case series. J Clin Neurosci.

[16] Myers LL, Nerminathan A, Fitzgerald DA, Chien J, Middleton A, Waugh MC, Paget SP (2020). Transition to adult care for young people with cerebral palsy. Paediatr Respir Rev.

[17] Seshadri SM, Rafaat KT, Brzenski A (2019). Anesthetic implications of duchenne muscular dystrophy and the surgical repair of scoliosis. Clinical Anesthesiology II.

[18] Lee JJ, Oh SH, Jeong YH (2020). Surgical strategies for cervical deformities associated with neuromuscular disorders. Neurospine.

[19] Marpole R, Blackmore AM, Gibson N, Cooper MS, Langdon K, Wilson AC (2020). Evaluation and management of respiratory illness in children with cerebral palsy. Front Pediatr.

[20] Ushirozako H, Hasegawa T, Yamato Y (2020). L5 pedicle subtraction osteotomy maintains good radiological and clinical outcomes in elderly patients with a rigid kyphosis deformity: a more than 2-year follow-up report. Eur Spine J.

[21] Ishihara Y, Morishita M, Kanzaki K, Toyone T (2020). Age-related progression of degenerative lumbar kyphoscoliosis: a retrospective study. Spine Surg Relat Res.

